# Measuring malaria by passive case detection: a new perspective based on Zambian experience

**DOI:** 10.1186/1475-2875-12-120

**Published:** 2013-04-10

**Authors:** Clive J Shiff, Cristina Stoyanov, Cornelius Choobwe, Aniset Kamanga, Victor M Mukonka

**Affiliations:** 1 Johns Hopkins Bloomberg School of Public Health, Baltimore, USA; 2 Malaria Institute at Macha, Choma, Zambia; 3 London School of Hygiene and Tropical Medicine, London, UK; 4 Copperbelt University, School of Medicine, Ndola, Zambia

**Keywords:** Malaria passive case detection, Rapid diagnostic tests, Zambia, Malaria incidence, Rural health centres

## Abstract

Most measurements of malaria are based on cross-sectional data and do not reflect the dynamic nature of transmission, particularly when interventions require timely data for planning strategies. Such data can be collected from local rural health centres (RHCs) where the infrastructure is sufficiently developed and where rapid diagnostics are in use. Because in rural areas, the population served by RHC is reasonably static, the regular use of malaria rapid diagnosis in RHCs can provide data to assess local weekly incidence rates, and such data are easily dispersed by cell phones. Essentially each RHC is a potential sentinel site that can deliver critical information to programme planners. Data collected during this process of passive case detection over a five-year period in the Macha area of Zambia show the importance of ecological zones and refugia in the seasonal fluctuation of malaria cases. If this process is implemented nationally it can assist in planning efficient use of resources and may contribute to local management and even elimination of malaria in this region.

## Background

Controlling malaria in Africa has been fraught with difficulties, not the least of these is the tenuous ability to sustain interventions and build on recent success. Scaling up malaria control interventions has been costly both in terms of commodities and personnel. Further, this approach has proved to be expensive to sustain, yet maintaining the gains attained over the years by any National Malaria Control Programme is critical to reducing further the disease burden in that country. Programme implementers now have to be innovative and begin to explore alternative cost-effective ways of tackling malaria, amidst dwindling financial resources from mainly external partners. This paper is a reflection on the situation in Zambia which marries active research programmes with the operation of a progressive and coordinated malaria control programme with the hope that operations can be extended to other endemic countries [1]. Currently extensive efforts are being made to reduce the impact of this disease in Zambia [2] and it is important to build on these successes by implementing currently available technologies efficiently and effectively to develop strategies that can focus on identifying reservoirs and on targeting vulnerable periods of the seasonal cycle of malaria transmission. Data collected in real time can help identify any such periods of the parasite population so these can be targeted in a cost-effective manner. A new way is needed to measure malaria in the field to help facilitate new strategies, and of the various parameters in use, the case incidence rate measured in real time is a useful tool.

**Figure 1:**
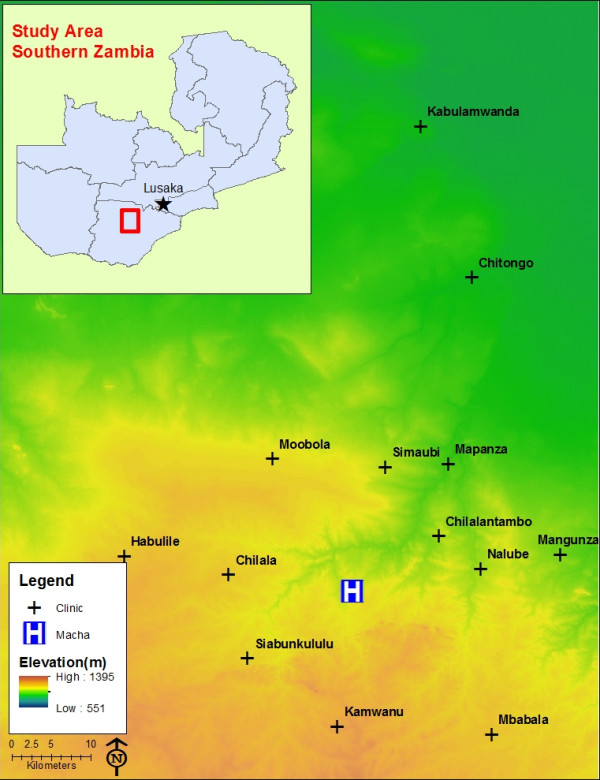
**Map of the Macha area in Southern Zambia.** Location of the rural health centres from which weekly malaria case incidence data was obtained. H is the location of Macha RHC as well as Macha Hospital and the Malaria Research Institute at Macha (MIAM). The box indicates the main ecological categories of the area. The zones referred to in Figure 2 are indicated: green being the flood plain of the Kafue River system with the higher, agricultural areas south of the large river system. Insert shows the extent of the flood plain and location of the various Rural Health Centres that report weekly case data.

**Figure 2:**
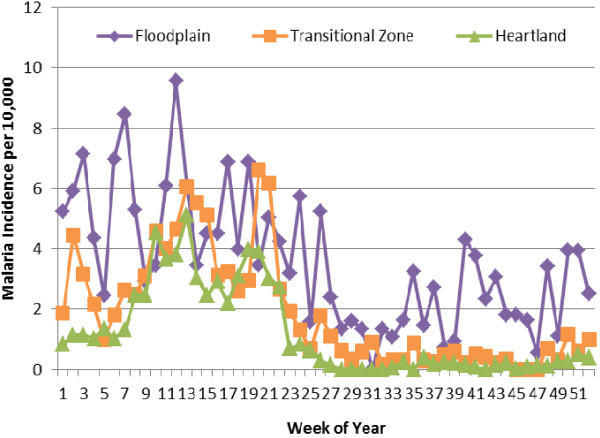
**Mean weekly incidence rate over three years recorded from RHC in the three ecological zones.** (after Davis *et al.*[5]) Refer to Figure 1 for illustration and layout of the zones [3].

Tom DeMarco’s influential 1986 book [3] “Controlling Software Projects: Management, Measurement, and Estimates” famously started with the aphorism that “you cannot control what you cannot measure”. This applies to malaria, as well as software implied in the above quote. However, with malaria it should follow with the caution that what is not measured accurately could be misleading. In spite of the seasonality associated with malaria transmission, collecting point prevalence data is still very much the *status quo*. Prevalence data are given per age group and are then annualized; data are usually collected from periodic blood film surveys and do not reflect the dynamic nature of the disease [4]. The need for a change of measurement was demonstrated by Ryan Davis who examined weekly case incidence data from a number of local rural health centres in the Macha area in Zambia (Figure 1). He showed not only how the weekly case incidence rate varied consistently over the annual season, but also how transmission was influenced by physical and geographical differences [5]. This can be seen in Figure 2. Malariometric methods are laid out in a protocol reviewed by Baird [6]. Passive case detection and active case reporting is the technique that has most relevance to control strategies, and it is now more feasible and instantly available with modern diagnostic procedures such as the malaria rapid diagnostic test (RDT). Used appropriately this technique can reflect the case incidence rate and thus the seasonal ebb and flow of transmission foci and thus provide evidence for targeting reservoirs of the parasite population when vulnerable to targeting [7]. In fact, it is suggested here that measuring malaria as a dynamic event is necessary to understand the impact of control interventions and should become an integral part of data-driven strategies for malaria elimination.

## Malariometric methodologies

### The malaria blood film test

The epidemiology of malaria as presently practiced is dependent on measurements that were devised over 100 years ago, *viz* the examination of Giemsa-stained blood films. Because of the nature of the process involved, it is difficult to maintain and use microscopes in the field, so there is usually a significant time delay in obtaining accurate and timely results. Realistically, in a rural centre, health post or clinic there is frequently a life-threatening delay in obtaining a diagnostic, hence the clinical “diagnosis” becoming the best option. However, the clinical diagnosis is very low on specificity, and this can lead to inappropriate drug administration, wastage of anti-malarial drugs and delays in investigating other possible ailments with symptoms that mimic malaria. There is also a culture of doubt in the treatment of malaria when point-of-care tests are used in the field [8].

Although it has some deficiencies, slide positivity is specific and can be used a measure of prevalence. However, to be meaningful epidemiologically the measurement is frequently extrapolated to estimate an annual figure, *viz* the annual parasite rate (APR) or API. APR or API are not rates, nor are they an expression of the annual transmission pattern of the parasite. Often this is all the information that is available, but it refers back to De Marco’s quote above. If one is attempting to determine a measure of parasite expansion, or distribution from one locality to another, the APR can be quite misleading. More sensitive data are needed most certainly as efforts to reduce or eliminate malaria disease in a sustainable manner are being considered. It is necessary to know the seasonal patterns of transmission and to follow the seasonal peaks and troughs as they happen. This is clearly demonstrated by Davis [5] in his study from southern Zambia (see Figure 2). The issue addressed here is how to collect and interpret the data available. The major advance in diagnostics came with the rapid diagnostic test for malaria [9].

### The rapid diagnostic test for malaria

In spite of many detractors, this point-of-care diagnostic test has revolutionized much of what is known about malaria. Certainly it has rationalized the use of anti-malarial drugs, but it has also contributed a new set of data relevant to transmission of malaria, data that are available at any point-of-care and in real time.

There are many different versions of the malaria rapid diagnostic test (RDT) based on histidine rich protein-2 (HRP-2) or lactose dehydrogenase (LDH) and whether the test is pan-malaria or specific for *Plasmodium falciparum.* The point here is that whatever test is used, the clinician has access to a diagnosis in real time. For the diagnostician, this is important as it permits a basis for appropriate and immediate treatment to save lives. For the epidemiologist, each diagnosis provides data from a known location that can also be available in real time, a huge advantage over the malaria blood film for which this information is seldom available. Data collected in a rural setting are easily dispersed to other parties regularly and in real time [10], something previously unavailable to the malariologist. This provides timely data to assess the local case incidence rate in weekly or other periodic frequencies. It is necessary for the RDT to be available for use in all health centres [11]; it is affordable and is much more than a simple method for diagnosis as it provides information on where and when the diagnosis was made. This information can be useful for planning interventions. The information it yields is of immediate epidemiological importance at miniscule extra cost, but to take advantage of this does entail a scientific upgrade of personnel in the health system.

### Malaria surveillance in real time: monitoring the incidence rate at the rural health centre, an example from Zambia

The potential value of this new data set requires a conceptual re-orientation in the staffing of the local health service, one from the Alma Ata concept of grass roots health care to a more scientific, data-driven enterprise. This will help develop strategic, evidence-based interventions with an eye to local sustainability. The rural health centre (RHC) will become a functional “sentinel site” and as these are spread over an entire country they can be a key source of important disease-specific data. With reference to malaria, the weekly disease positivity rate is essential. To collect and use this information, local staff will need training in data collection, data validation and some basic epidemiology to mobilize the information. The purchase of a mobile phone is a one-time investment and cost reimbursement for text time or sending SMS is quite affordable [10]. The approach requires no intensive training and is simple to conduct, thus curtailing the costs associated with the traditional implementation of vector control and treatment interventions. At district and provincial levels, professional staff will require specific training in epidemiology in order to collect and validate weekly data and perform basic and analytical tasks for use at national level to plan and operate targeted interventions. These stages will require specialist training in geographical information system approaches and this analytical approach will enable better and more efficient planning, use of commodities and evidence-based programme implementation. The data will further contribute to better planning for an impending seasonal outbreak by ensuring that logistics are procured and distributed well in advance. However, to calculate an incidence rate requires a denominator, and in reasonable stable population groups such as occur in agricultural areas, this is feasible [5].

The RHC and clinic is the fundamental link between the Ministry of Health and the community in rural Zambia, and for that matter in most parts of the developing world. The centres are staffed by trained personnel and, depending on the government policy, are stocked with supplies, drugs and materials to serve the primary health needs of the community. Apart from the natural increase, in Zambia estimated at 3% annually, in rural areas the population is reasonably stable; for the most part the public is drawn from farmers and the farming community. People generally live on their farms. Travel occurs, but major migrations are seldom, and where this happens seasonally health personnel are well informed. For handling the malaria situation, health workers are generally supplied with essentials. RDT and appropriate drugs are kept in stock for the most part, although stock-outs do occur. Diagnosis and treatment are provided free-of-charge, and treatment should only follow positive diagnosis. The compliance to current treatment regimen is clear and is monitored. Records are made of all treatments and are entered into the clinic register. Clinic staff members are informed of the local demographics and have estimates of the number of persons served. These estimates are based on census data or on information from the local authorities. This provides a reasonable population estimate that is used for a denominator so as to convert the weekly case-load to an incidence rate that can be used to integrate data into a district or provincial database for further analysis.

Communication with the health centres has been greatly expedited since mobile phone systems were introduced some five years ago and the majority of the country is now connected by mobile phone networks [10]. This advance now enables each RHC to operate as a timely source of data. Thus the RHC is part of a nationwide data system that can impart information to a central authority on an as-needed basis. This is important for epidemiological analysis when the data are best expressed in the Julian calendar format, i e, a 52-week year. In this way, week 40 in year 1 can be compared with week 40 in year 2, etc. The ability to quote a figure of case incidence per specific seasonal week is extremely powerful and a new vital statistic that can be used by the national planners. An example of the system at the RHC level is given in Figure 1 (map of the research area in Southern Zambia) and the graphs in Figure 2[5]. The map is divided by topography and elevation into three zones: flood plain (Kafue River basin), transitional zone, and heartland. All areas are settled and provide a basis for the agriculture of the area, although the flood plain zone is more pastoral than crop orientated. Transmission followed seasonal patterns of high transmission and periods of low or negligible transmission, and these patterns were consistent over five years of observation.

### Malaria risk assessment: an ecological-topographical analysis

It has long been understood that malaria often occurs as a focal disease, at least during parts of the annual cycle. Working in what was then Rhodesia in 1928, Leeson described “malaria houses” where residents appeared to be prone to infection [12]. Yet is has been difficult to ascribe factors consistent with high risk other than proximity to water and similar ecological conditions. Perhaps this is due to timing or a lack of attention to the seasonal patterns of malaria transmission. However in the example of this paper, certain discreet foci seem to occur in the flood plain zone during dry, low transmission periods and are likely to persist due to local conditions that permit mosquito foraging during periods of general aridity. The objective is to detect such situations and define the climatic and topographical characteristics associated with these foci if, in fact, there is any similarity between them. This will help to re-orientate the epidemiologist from concentrating on details of house structure and site locality to a more area-wide and landscape-wide approach in order to assess risk of infection.

**Figure 3:**
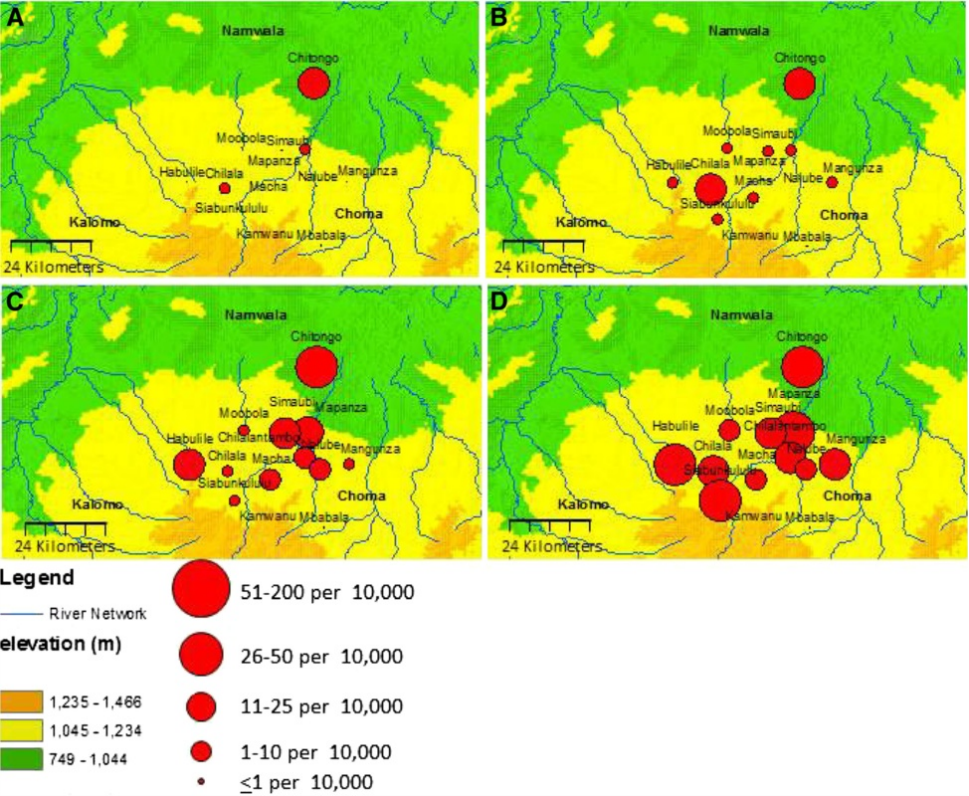
**Seasonal changes of the case incidence rate of malaria from each of the 13 rural health centres in the study area.** (**A**) shows mean weekly incidence rates for all RHC weeks 40–46 and (**B**) weeks 47–52 in 2008. (**C**) Represents weekly incidence rates during weeks 1–6 and (**D**) during weeks 7–12 in 2009. The figure shows the importance of the flood plain (green) area for refugia of the parasite population during the hot-dry season.

**Figure 4:**
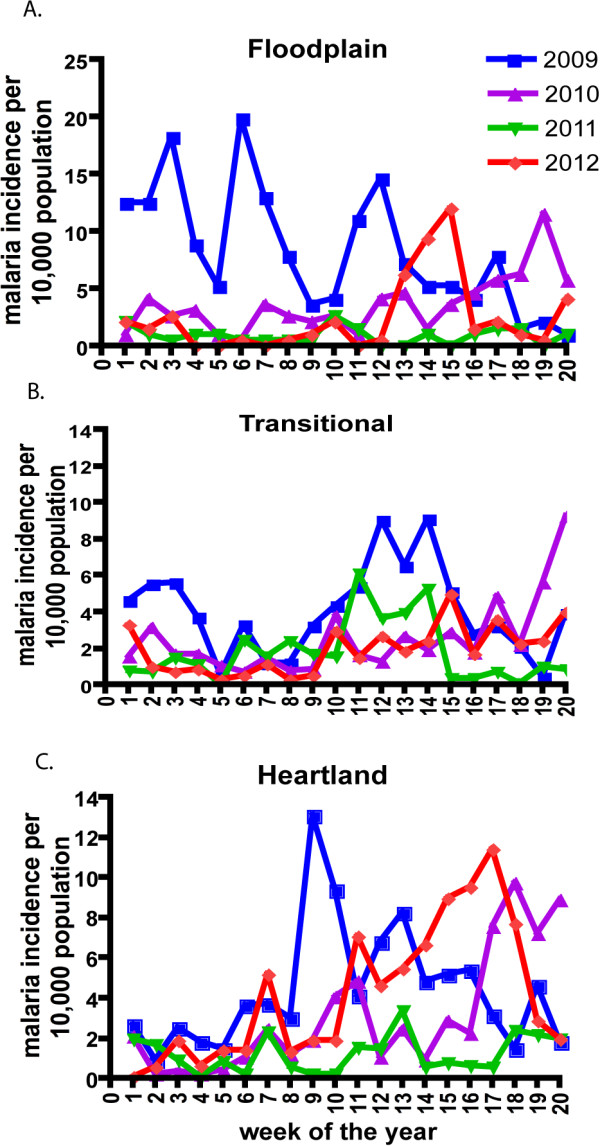
**Mean weekly case incidence rates in the three topographical zones in the Macha research area for the years 2010–2012 during the peak transmission period.** Cool weather in May (weeks 20 onwards) have a depressing effect on transmission and hence the case incidence rate and are omitted to avoid cluttering of the figures. Note ordinate scales for floodplain differ from that of the other two localities.

In the Macha study area, weekly case incidence rates at RHCs were used to help identify transmission foci. Each RHC represents a focal “catchment” and the overall transmission patterns week by week within that community. The general landscape can be assessed from satellite imagery (Figure 1) and data provide a clear pattern of the transmission pattern throughout the year (Figure 2). This gives proximal knowledge of the areas of risk, not only at peak transmission when malaria is ubiquitous, but also at times of low transmission when that foci become discernible and critical bottlenecks can be observed and predicted (Figure 3). Targeted interventions can then be mounted. Bearing in mind that the RDT is unlikely to be positive with asymptomatic infections and thus may miss many gametocyte carriers, such information still suggests topographical areas of risk within the patient catchment area, both in time and place. These data (Figures 3 and 4) come from five consecutive years of continuous observation.

### The importance of topography and season

The malaria case incidence has been recorded from 14 RHCs for each calendar week since August 2008 (the second site in the flood plain, Kabulamwanda was incorporated in the study in 2010 and is not shown in Figures 1 and 3). The patterns of transmission in the various topographical zones (Figure 2) are discernible and define the relative importance of the overall topography each season. Two centres are in a flood plain ecosystem, a well-watered, somewhat homogeneous zone of grassland with a fairly high water table approximately 2–4 m deep. There are four RHC in the “transitional” zone that is more elevated in hilly ranges with shrub and small tree vegetation and numerous agricultural lands. The remainder are in the ecological heartland, an area of general agriculture and settlement [5] but where the water table is some 20 m deep. Data points in Figure 2 are a mean of three annual readings: 2008, 2009 and 2010 and show that there is a regular five-month period (June-October) when the occurrence of cases within the whole study area is confined mainly to the flood plain environment. This is apparently a bottleneck in the parasite population and will perhaps be vulnerable to elimination and can be seen more clearly in Figure 3 (A-D). During the period 2008–9 there was widespread distribution and use of insecticide-treated bed nets which affected transmission of malaria in the heartland and transitional zone. Data in Figure 4 show the result of this intervention in the three topographical zones during 2010, 2011 and 2012. Perusal of Figure 4, where the mean weekly incidence of malaria is shown for weeks 1–20 for 2009, 2010, 2011 and 2012, indicates a discernible difference in the incidence rates between 2009 and 2012 early intervention and three years post intervention, from the 2010 and 2011 seasons when some reduction in transmission was evident. One could postulate this was due to a decline in the insecticidal efficacy of the intervention which commenced in mid-2008 [13]. This effect is seen best from week 1–10 in all three ecological zones. Looking at the patterns of transmission (Figures 2 and 3) during the period weeks 30–46, there was evidence of sustained but low level of transmission throughout the five-month dry and hot period each year in the floodplain only. Sporadic cases occurred in the transition area but almost no cases presented in the clinics of the heartland. The patterns suggest that the cases detected in the flood plain during the low transmission period represent local hot spots or reservoirs, and that these foci maintain the residual parasite population to infect the next generation of mosquitoes which then spread throughout the area.

### Data analysis: importance of timing and detection of parasite reservoir

The focality of malaria, particularly during periods of low transmission, is an important factor to be understood when planning interventions, hence the importance of collecting data that can reveal this. Yet in most studies on hot spots, data are collected through the entire year and presented as averages. At the height of the transmission season when malaria is more or less ubiquitous, any focality is lost in the plethora of disease. When the foraging vectors are not under stress it is difficult to assign points of structure, situation of houses or environmental conditions that will act as risk indicators or even the presence of hot spots. But it was shown in Zambia that conditions are critical when the vector population is under stress; this population is depleted or killed off by cold weather, and foraging behaviour becomes highly restricted by high temperatures and arid conditions by day or night (personal communication, Stoyanov *et al.,* 2013). At times of high transmission attempts to define factors associated with risk of infection are hard to interpret [14], other than by looking at the overall topography and landscape and the results of passive case detection carried out by the local health personnel through RHCs, see (Figure 3D). Things change following the onset of cold conditions after May to June when the vast proportion of cases detected is concentrated in the flood plain of the Kafue River. The implication is that at this time and until the onset of the rains, mosquito vectors are able to forage and sustain various foci of infections in limited ecologically suitable foci that sustain the parasite population. It is an important conclusion that the foci represent a specific type of habitat typified by a riverine flood plain, as this environment provides the conditions that permit transmission.

### Lessons learned

Observations carried out over a period of five years in the Macha area provide a basis for following the case incidence rate in different landscape situations. The ebb and flow of cases detected passively in RHCs in the flood plain, transitional zone and heartland (Figure 3A-D) in 2008–09 show clearly where the reservoir parasite population is located during the season of stress for mosquito foraging, and how it expands progressively with the onset of the rains in November until April by which time malaria is occurring throughout the entire study area. This pattern has been repeated every year since 2008. It is appropriate to list some lessons learned:

1. The parasite population is restricted to the flood plain habitat during the period June to November, mainly because this is the only ecosystem where winter cold does not kill off the main vector population, and where there is sufficient nocturnal humidity to permit foraging by mosquitoes. This sustains the local hot spots;

2. While the RDT does not identify all asymptomatic cases of malaria, the case incidence rate per week reveals the seasonal patterns of transmission and the location of such hot spots in space and time;

3. The study promotes the usefulness of information at the point of collection, it is a grass-roots process where treatment and data collection can be integrated and presented in a time-sensitive manner to central authorities for analysis. Unlike the current practice of having central level driven interventions based on aggregated and averaged data, the study provides information and data specific to the catchment population which is important in understanding the epidemiology and application of specific interventions to be timely and have an impact.

## Competing interests

The authors have declared that they have no competing interests.

## Authors’ contributions

The overall concept was developed by CJS with CS participating in the write-up and data management. AK and CC gathered and checked data sent weekly from the RHC staff and followed up periodically to validate the information and provide feedback to the personnel involved. VMM, as Scientific Director in the Ministry of Health, was instrumental in upgrading the conditions in the rural health system in Zambia and thus enabled the study, he also contributed to the write-up. All authors read and approved the final manuscript.
